# Ecotypic Identity and Manipulated Rainfall Modulate Diversity‐Productivity Relationships Across a Precipitation Gradient

**DOI:** 10.1002/ece3.73464

**Published:** 2026-04-09

**Authors:** Zhe Ren, David J. Gibson, David F. Barfknecht, Sara G. Baer, Matthew B. Galliart, Jack R. Sytsma, Loretta C. Johnson

**Affiliations:** ^1^ Department of Biological and Clinical Sciences University of Central Missouri Warrensburg Missouri USA; ^2^ Department of Botany and Plant Pathology Purdue University West Lafayette Indiana USA; ^3^ School of Biological Sciences Southern Illinois University Carbondale Illinois USA; ^4^ Department of Forest and Wildlife Ecology University of Wisconsin Madison Wisconsin USA; ^5^ Department of Ecology & Evolutionary Biology and Kansas Biological Survey & Center for Ecological Research University of Kansas Lawrence Kansas USA; ^6^ Department of Biological Sciences Fort Hays State University Hays Kansas USA; ^7^ Division of Biology Kansas State University Manhattan Kansas USA

**Keywords:** *Andropogon gerardi* Vitman, ecotype, generalized boosted regression trees, generalized linear mixed model, tallgrass prairie, US Great Plains

## Abstract

Understanding the context‐dependent nature of diversity‐productivity relationships is essential for predicting community responses to environmental change and informing restoration strategies. We conducted a reciprocal common garden experiment across a rainfall gradient (mean annual precipitation: 500–1200 mm year^−1^) in the US Great Plains to examine how local ecotypes of dominant grasses [e.g., *Andropogon gerardi* Vitman and *Sorghastrum nutans* (L.) Nash] and experimental drought (rainout manipulation) shape diversity‐productivity relationships. We applied generalized linear mixed models to assess the effects of aboveground live biomass, seed source, and rainfall manipulation on grassland species richness, phylogenetic diversity, and functional diversity. Functional diversity was positively correlated with aboveground live biomass at the dry site. Dominant species ecotypes influenced diversity‐productivity relationships in a site‐specific manner. The DRY ecotype reduced species richness as aboveground live biomass increased at the driest and mesic sites while the MESIC‐ecotype increased phylogenetic diversity with rising aboveground live biomass at the mesic site, with no effects observed on functional diversity. We further applied generalized boosted regression trees to assess the relative importance of aboveground live biomass, seed source, and rainfall manipulation in shaping grassland diversity. Across all sites, aboveground live biomass was the strongest predictor of diversity, whereas the effects of seed source and rainfall were weaker and more variable, likely reflecting the dominant role of locally adapted grasses in structuring communities and the limited translation of rainfall manipulation into consistent compositional change. These findings highlight the central role of dominant species attributes in shaping diversity‐productivity relationships and stress the importance of considering local adaptation and site context in restoration and biodiversity‐function research.

## Graphical abstract

Understanding how diversity‐productivity relationships vary across environmental contexts is critical for predicting grassland responses to climate change and guiding restoration. Using a reciprocal common garden experiment across a Great Plains rainfall gradient, we found that aboveground live biomass was the strongest predictor of species, phylogenetic, and functional diversity, with effects mediated by site conditions and dominant grass ecotypes. Local adaptation of dominant species shaped diversity‐productivity patterns in a site‐specific manner, underscoring the importance of context and dominant species traits in biodiversity‐ecosystem function research.

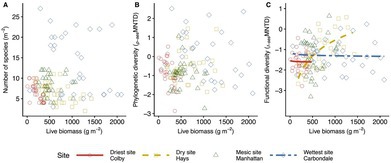

## 1 Introduction

Deciphering the complexity of the diversity‐productivity relationships remains a key ecological challenge with significant relevance to nature‐based climate solutions (Mori et al. 2021; Andraczek et al. 2024). One of the widely recognized patterns, the unimodal humped‐back model (HBM) proposed by (Grime 1973), predicts that species richness peaks at intermediate levels of productivity (Al‐Mufti et al. 1977). This HBM relationship, with productivity treated as a predictor of diversity, has been widely used to explain grassland diversity patterns across environmental gradients from regional to global scales (Fraser et al. 2015). Although the HBM has broad global support (Fraser et al. 2015), its applicability is context‐dependent (Catford et al. 2022), influenced by factors such as land use and land cover history on the species pool (Adler et al. 2011), aridity (Rey et al. 2016), sampling biases (Oksanen 1996), nutrient limitation (Palpurina et al. 2019), and other abiotic drivers including disturbance (Li et al. 2021; Ónodi et al. 2021) and successional stage (Guo 2003). Beyond the unimodal pattern, many studies have documented either a positive (Cardinale et al. 2006; Chiarucci et al. 2006; Liang et al. 2016; Barry et al. 2020; van Ruijven et al. 2020) or negative (Grace et al. 2007; Símová et al. 2013; Andraczek et al. 2024; Bai et al. 2024) relationship between diversity and productivity, indicating that non‐unimodal patterns are also frequently observed in plant communities. One potentially overlooked factor contributing to this variability in diversity‐productivity relationships is the role of local adaptation in dominant species that shape patterns of community assembly (Michalet et al. 2006). Local adaptation in dominant species can modify environmental resource and stress regimes, thereby influencing which species persist within communities. In turn, the identity of dominant species and their mass‐ratio effects, whereby the traits of the most abundant species disproportionately drive ecosystem functioning, play a central role in linking patterns of diversity with productivity (Grime 1973; Avolio et al. 2019; Smith et al. 2020).

To fully understand the context dependency or generality of diversity‐productivity relationships, it is important to examine diversity metrics beyond taxonomic diversity. Although diversity‐productivity relationships have been widely studied using species richness across taxa (Laanisto et al. 2008; Kessler et al. 2014), additional insight can be gained by incorporating other dimensions of diversity, collectively known as “attribute diversity” (Chalcraft et al. 2009; Chao et al. 2014; Brun et al. 2019). Phylogenetic diversity captures evolutionary relationships among species (Cavender‐Bares et al. 2006; Barber et al. 2017; Khalil et al. 2017; Cadotte et al. 2019), while functional diversity reflects differences in species traits relevant to ecosystem functioning (Petchey and Gaston 2002; Spasojevic and Suding 2012; Purschke et al. 2013; Khalil et al. 2018; Miller et al. 2019). Like species richness, phylogenetic and functional diversity offer distinct perspectives on species dominance and similarity in communities (Ren et al. 2023) and help explain variation in productivity (Cadotte et al. 2009). For instance, some studies highlight a strong association between phylogenetic diversity and aboveground live biomass, as phylogenetic diversity often reflects broad variation in functional traits because evolutionary history constrains and structures trait variation among species (Cadotte et al. 2009; Flynn et al. 2011; Jia and Wang 2024). However, others caution that using phylogenetic diversity as a proxy for functional diversity may lead to biased interpretations, as functional diversity captures unique trait‐based differences that are not always reflected in evolutionary relationships, particularly when species richness is constant (Steudel et al. 2016; E‐Vojtkó et al. 2023).

An ecotype is a locally adapted population defined by heritable phenotypic and genetic traits that have been shaped by natural selection under specific environmental conditions (Gregor 1944; Hufford and Mazer 2003). Among dominant species, those exhibiting ecotypic variation can maintain dominance across regional environmental and productivity gradients by adapting to local conditions (Turesson 1922; Lowry et al. 2014). The genetic variations in dominant species, specifically the emergence of ecotypes, can strongly shape community structure (Whitham et al. 2020; van Zuidam et al. 2019). If ecotypic variation in dominant species alters diversity‐productivity relationships, then the source of plant material used in restorations may substantially affect the composition and functioning of reassembled communities (Baer et al. 2019). Ecotype selection, therefore, plays a critical role in restoration outcomes.

One of our focal species, big bluestem ( *Andropogon gerardi* Vitman), is ideal for testing the effects of dominant grass ecotypes on the diversity‐productivity relationship (Epstein et al. 1997; Johnson et al. 2015; Gibson et al. 2016; Galliart et al. 2020). For instance, *A. gerardi* maintains dominance across the tallgrass prairie ecosystem and exhibits distinct local ecotypes adapted to regional gradients in temperature and rainfall (McMillan 1959; Gustafson et al. 1999, 2004; Gray et al. 2014; Galliart et al. 2019). However, little information exists on the ecological responses of the co‐dominant native North American prairie species Indiangrass ( *Sorghastrum nutans* Nash), likely because research on tallgrass prairies has disproportionately focused on the dominant species *A. gerardi* . While prior studies have shown that ecotypic variation can influence interspecific interactions (Liancourt and Tielbörger 2011) and that intraspecific variation in dominant species can affect community diversity (Gibson et al. 2012; Klopf et al. 2014), it remains uncertain how local ecotypes of dominant species influence diversity‐productivity relationships. Clarifying the role of dominant species in diversity‐productivity relationships will inform understanding and projections of climate‐mediated community shifts (Polley et al. 2007; Sasaki and Lauenroth 2011; Johnson et al. 2015).

In temperate grasslands, productivity is commonly estimated using aboveground live biomass (Gillman and Wright 2006). Typically, such estimators include either cumulative annual aboveground biomass (Junk and Piedade 1993) or peak‐season biomass (Fraser et al. 2015). Indirect indicators such as environmental variables (e.g., rainfall, temperature, or evapotranspiration; Símová and Storch 2017) and biotic factors, including ecotypic differentiation (Fetcher and Shaver 1990; Mendola et al. 2015; Stahlheber et al. 2020), may also act as proxies for functional variation that influences biomass production, although they are not direct measures of productivity (Lisner et al. 2021).

We aimed to determine whether diversity‐productivity relationships are consistent across a rainfall gradient by testing how aboveground live biomass, dominant grass ecotypes, and rainout‐simulated drought influence restored U.S. tallgrass prairie diversity metrics. Specifically, we analyzed diversity‐productivity relationship patterns using a reciprocal common garden experiment, incorporating a reduced rainfall treatment, reciprocally sown with putative ecotypes of co‐dominant grasses (Silletti and Knapp 2002) replicated across a 681 mm year^−1^ rainfall gradient. Incorporating a rainout treatment into the common garden experiment allows direct comparison between ambient and reduced rainfall conditions, as previous studies show that severe experimental drought can reduce species diversity and accelerate shifts in grassland community structure by causing the local extinction of subordinate species (Tilman and El Haddi 1992; Smith et al. 2020; Knapp et al. 2024). Ecotypes of dominant species were used in the common garden experiment to represent populations adapted to different rainfall conditions, allowing the experiment to test whether grassland community responses to rainfall are driven by genetic differences rather than local growing conditions. The dominant grass species were sourced as seed (hand‐collected) from three different regions across a steep rainfall gradient (Johnson et al. 2015, 2022). We asked three questions:

Question 1: Does the relationship between diversity (taxonomic, phylogenetic, or functional) and productivity differ among sites along a rainfall gradient spanning mean annual precipitation from 520 to 1201 mm year^−1^, and between rainout shelter treatments? We expected the unimodal diversity‐productivity relationship, as shown globally in grasslands (Fraser et al. 2015), to shift toward lower peak productivity at drier sites or under rainout shelters compared to wetter sites or without rainout shelters (Cherwin and Knapp 2012), though the intraspecific response of grassland dominants to drought and their role in community resilience are not clearly understood currently (Weißhuhn et al. 2011; Jung et al. 2014; Miller et al. 2019; Ren et al. 2024).

Question 2: How do different ecotypes of dominant species affect diversity‐productivity relationships? We predicted that locally adapted ecotypes would exhibit higher productivity when grown in their home habitats (home‐site advantage; Joshi et al. 2001), while reducing community diversity compared to non‐local ecotypes.

Question 3: What are the relative contributions of key predictors (aboveground live biomass, ecotype source, and rainout shelter treatment) to explaining plant diversity at each site? We expected diversity in high‐biomass grasslands to be constrained by competitive exclusion, because increasing biomass intensifies competition for light in grassland and reflects realized dominance more directly than indirect indicators such as seed source or rainfall treatment (Grime 1973; Lisner et al. 2021; Johnson et al. 2015).

## 2 Materials and Methods

### 2.1 Study Sites

**FIGURE 1:**
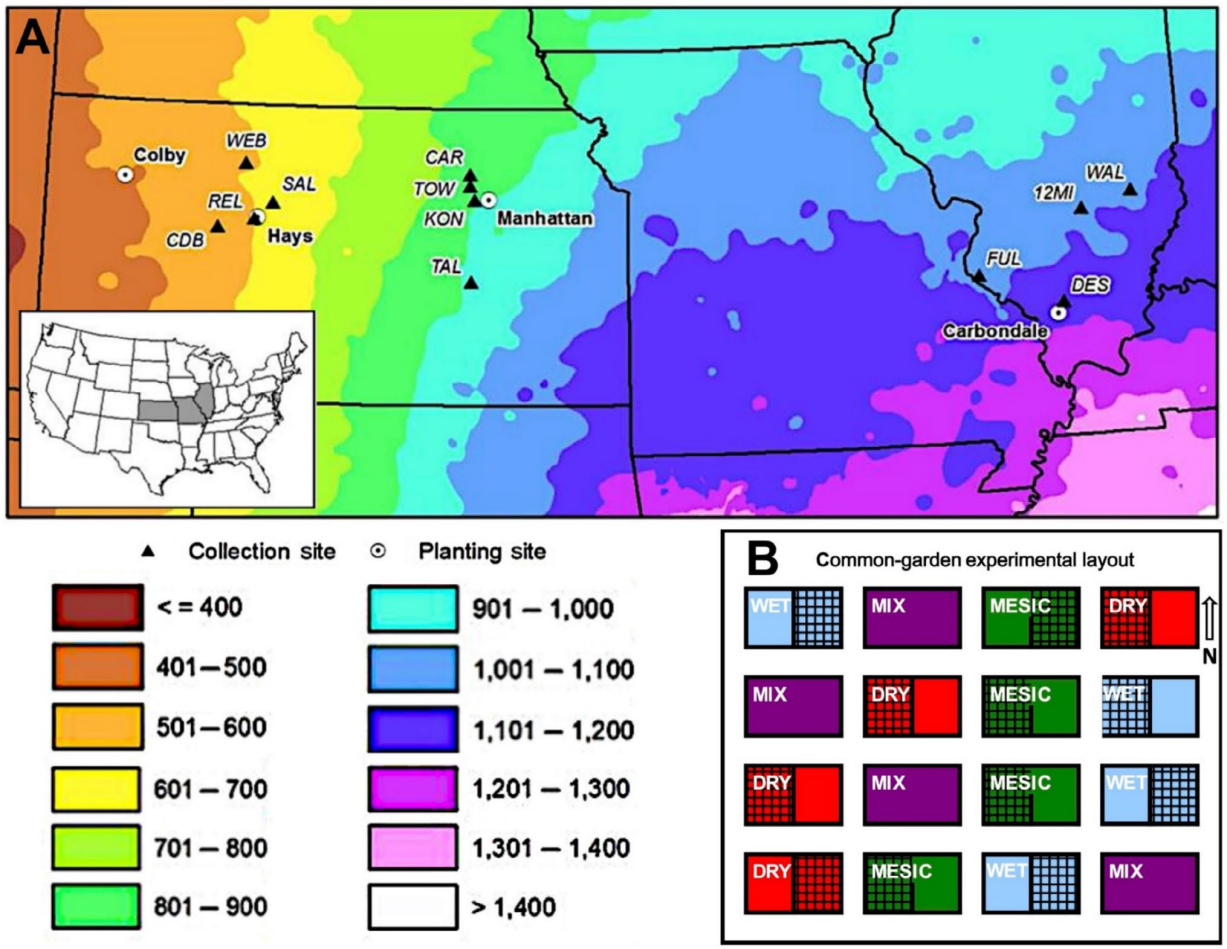
Illustration of research sites and experimental design: (A) Map showing location of reciprocal garden sites (Colby, Hays, Manhattan in Kansas, and Carbondale in Illinois) and seed collection sites across the US Great Plains. Modified from Galliart et al. (2019). Colors indicate mean annual precipitation (mm year^−1^) (1981–2020); (B) Schematic of the common garden experimental design showing the spatial arrangement of WET (blue), MESIC (green), DRY (red), and mixed (purple) ecotype plots under ambient (solid) and rainout (grid‐patterned) rainfall treatments.

Reciprocal common gardens were established in 2009 at four sites: Colby, Kansas (KS; driest), Hays, KS (dry), Manhattan, KS (mesic), and Carbondale, Illinois (IL; wettest) (Figure 1A). These sites experienced an increase in mean annual precipitation (MAP) from 520 mm year^−1^ (driest site) to 1201 mm year^−1^ (wettest site; 30‐year averages) over 1000 km from west to east across the US Great Plains. The sites had previously been in agricultural production and were prepared by light disking in the fall of 2008. While full descriptions of the reciprocal transplant gardens and their establishment are available in previous studies (Wilson et al. 2016; Galliart et al. 2019; Johnson et al. 2015, 2022), a summary is provided below. Comparison of results across all sites from the four reciprocal transplant gardens allowed for a regional scale comparison.

### 2.2 Seed Sources of Dominant Species

Big bluestem ( *Andropogon gerardi* Vitman) and Indiangrass [ *Sorghastrum nutans* (L.) Nash], the two dominant species in this study, were used to establish experimental plots. *A. gerardi* seeds were collected in the Fall of 2008 from remnant prairies within an 80 km radius of the dry, mesic, and wet garden sites, while *S. nutans* seeds were collected from four remnants near the wettest site and one near each of the dry and mesic sites (Johnson et al. 2015). Seeds of eight non‐dominant prairie species (Appendix S1) were purchased from a commercial supplier (Ion Exchange Inc., Harpers Ferry, Iowa, USA). Seeds were kept in dry storage before planting. *A. gerardi* seed sources used in this study represent regionally defined ecotypes (DRY, MESIC, and WET) that are genetically differentiated and exhibit consistent functional trait differences, and variations in growth, fitness, tissue chemistry, and forage quality (Epstein et al. 1997; Olsen et al. 2013; Caudle et al. 2014; Gibson et al. 2016; Galliart et al. 2020; Systma et al. 2026; Appendix S2). These genetically and functionally distinct ecotypes have been shown to differentially influence biodiversity‐ecosystem function relationships across multiple community‐ and ecosystem‐level responses (Johnson et al. 2008; Wilson et al. 2016; Galliart et al. 2020; Ren et al. 2023). In our long‐term experiment, pronounced genetic differentiation among ecotypes (Gray et al. 2014), coupled with strong and persistent trait divergence (Johnson et al. 2015), was evident by 2010 and remained stable through 2016 (Galliart et al. 2019). Although ecotypic variation in *S. nutans* remains understudied, prior research suggests patterns similar to those in *A. gerardi* (McMillan 1959; Gustafson et al. 2004; Baer et al. 2014). In 2021, phenotypic differences among *S. nutans* ecotypes were quantified for key functional traits, including specific leaf area, maximum mature height, seed mass, and leaf nitrogen content (Ren et al. 2023).

### 2.3 Garden Design

At each site, four blocks were established, each containing four randomized plots seeded with either a regional ecotype of *A. gerardi* and *S. nutans* (DRY, MESIC, or WET) or a mixture (MIX) of all three ecotypes (Figure 1B). This design enabled testing of both site and ecotypic effect on diversity. The MIX plots provided the opportunity to maximize the mitigation potential of ecotypes under future climate change through “composite provenancing” (Broadhurst et al. 2008; Johnson et al. 2015). Genetic analyses revealed that MIX plots were dominated by the DRY ecotype at the driest and dry sites 5 years after garden establishment but were comprised of a mixture of ecotypes at the mesic site and were dominated by the WET‐ecotype at the wettest site (Galliart et al. 2019).

Local ecotype and MIX plots measured 4 m × 8 m, while non‐local plots at each site were 4 m × 4 m. At the driest site (Colby, KS), all plots were 4 m × 4 m regardless of ecotype. In June 2009, plots were seeded with the regional ecotype of *A. gerardi* (270 live seeds m^−2^) and *S. nutans* (70 live seeds m^−2^), along with eight additional non‐dominant species at a total density of 580 seeds m^−2^, following restoration guidelines in the Great Plains region (Diboll 2005). Plots were left unweeded to allow establishment of volunteers from the regional species pool. Plots were separated by 4–6 m buffer strips seeded with sideoats grama [ *Bouteloua curtipendula* (Michx.) Torr.] and little bluestem [ *Schizachyrium scoparium* (Michx.) Nash], sourced from a commercial supplier (Ion Exchange Inc., Harpers Ferry, Iowa, USA).

To assess the effects of reduced rainfall on diversity, rainout shelters were installed in Spring 2011 at the dry (Hays, KS), mesic (Manhattan, KS), and wettest (Carbondale, IL) sites. Shelters were placed over half of all plots, excluding the MIX plots. Following the design by Yahdjian and Sala (2002), each shelter consisted of clear acrylic *V*‐shaped plates (2.00 m × 1.88 m), 2 m long and 0.13 m wide, spaced 20 cm apart and angled at a 20° slope. At the Kansas sites, runoff was collected in barrels and discarded away from the plots; in Illinois, rainwater was diverted using a gutter on the low side, with the outlet positioned away from the plot. The lowest side was elevated 1.5 m above ground level to avoid intercepting vegetation. Shelters were installed annually from late May to early June, after 25% of growing degree days had accumulated at each site. Designed to intercept 50% of ambient precipitation, the shelters reduced rainfall by 34%–38% beneath the structures, resulting in soil moisture reductions of 8%, 14%, and 25% by 2012 (Wilson et al. 2016), and 14.6%, 22.8%, and 21.3% during the 2021 season (J.R. Sytsma, unpublished) at the dry, mesic, and wettest sites, respectively.

### 2.4 Vegetation Survey

Diversity and productivity data were collected in 2012 and 2021, corresponding to three and 12 years after the establishment of the common garden experiment. In late summer of each year, observers visually estimated canopy cover of vascular plants rooted within four 1‐m^2^ quadrats per plot, using it as a proxy for species abundance. The maximum cover value for each species across quadrats was retained for analysis, and quadrat data were averaged at the plot level.

Consistent with previous studies (Al‐Mufti et al. 1977; Guo and Berry 1998; Adler et al. 2011; Fraser et al. 2015), we used peak‐season aboveground live biomass as a proxy for productivity because it closely approximates annual aboveground net primary productivity in grasslands (Ghorbani et al. 2020), integrates cumulative growing season carbon gain (Sala and Austin 2000), and captures climatic controls on plant production (La Pierre et al. 2011). Productivity was measured in late Summer of 2012 and 2021 by harvesting aboveground live biomass rooted within four randomly placed 0.1‐m^2^ quadrats (50 cm × 20 cm) per plot. All standing plants were clipped at ground level, bagged in the field, oven‐dried at 60°C for 48 h, and then weighed. Litter, including woody shrub stems, was excluded due to difficulty in distinguishing current‐year from previous‐year material, which could otherwise inflate productivity estimates (Hassan et al. 2021).

### 2.5 Phylogeny

Based on plant community surveys across all sites, maximum likelihood phylogenies were constructed in 2012 and 2021 to include all taxa recorded in the sample plots (Appendix S3). Nucleotide sequences for *rbc*L and *mat*K were retrieved from GenBank (Benson et al. 2013; ncbi.nlm.nih.gov/genbank/). Sequences were aligned individually using MUSCLE (version 3.8.1551; Edgar 2004) in SeaView (version 4.0; Gouy et al. 2010), trimmed, and concatenated using Mesquite (version 3.70; Maddison and Maddison 2021). Phylogenetic inference was performed using the W‐IQ‐TREE web server (Trifinopoulos et al. 2016; iqtree.cibiv.univie.ac.at/), with model selection based on AIC, AICc, and BIC criteria.

### 2.6 Functional Traits

For each species, we measured five continuous functional traits: specific leaf area (cm^2^ g^−1^), maximum vegetative height (cm), leaf area (cm^2^), leaf nitrogen content (mg g^−1^), and seed mass (g 1000 per seeds); and five categorical traits: growth form (e.g., graminoid, legume, or non‐legume forb), life span (annual, biennial, or perennial), pollination mode (biotic or abiotic), seed dispersal vector (biotic or abiotic), and clonal reproduction (present or absent). These traits were selected to capture key dimensions of plant fitness, including resource acquisition, reproduction, and persistence (Pérez‐Harguindeguy et al. 2013). Functional trait values for each species were obtained through standardized measurements following established protocols (Cornelissen et al. 2003), using data from Khalil et al. (2019), Ren et al. (2023), or retrieved from the TRY Plant Trait Database (Kattge et al. 2020). Functional trait dendrograms were constructed in 2012 and 2021 to illustrate the functional similarity among species (Appendix S4).

### 2.7 Diversity Metrics

To investigate diversity‐productivity relationships, we considered a range of diversity indices, including species richness, phylogenetic and functional trait‐based metrics (Tucker et al. 2017). Correlations between diversity indices and productivity were assessed using the *corrplot* function in the “corrplot” R package (version 0.95; Wei and Simko 2021; Appendix S5). We focused on estimating mean nearest taxon distance (MNTD) for phylogenetic and functional diversity respectively using the *ses.mntd* function in the “picante” R package (version 1.8.2; Kembel et al. 2010) because (1) it has a relatively low Type I error rate (Miller et al. 2017), and (2) it captures fine‐scale evolutionary or functional pattern, reflecting ecological processes such as environmental and biotic filtering (Massante et al. 2021). MNTD is considered a terminal phylogenetic metric, estimating the mean phylogenetic distance between each species and its nearest relative within the community (Swenson 2014). To account for the influence of species richness on phylogenetic and functional diversity (Miller et al. 2017), we calculated abundance‐weighted standardized effect sizes of MNTD as:

$$\text{sesMNTD}=\frac{{\text{MNTD}}_{\text{observed}}-{\text{MNTD}}_{\text{null}}}{\mathrm{SD}\left({\text{MNTD}}_{\text{null}}\right)}$$

where ${\text{MNTD}}_{\text{observed}}$ is the observed mean nearest taxon distance in the community, ${\text{MNTD}}_{\text{null}}$ is the mean of expected MNTD values from 1000 randomizations of the community, and $\mathrm{SD}\left({\text{MNTD}}_{\text{null}}\right)$ is the standard deviation of those null values. Negative sesMNTD values (*p‐*sesMNTD and *f‐*sesMNTD, respectively) indicate that species in the community are more evolutionarily or functionally clustered than expected by chance (e.g., low phylogenetic or functional diversity), while positive values indicate that species are more distantly related than expected (e.g., high phylogenetic or functional diversity).

### 2.8 Data Analysis

To address Question 1 and 2 on diversity‐productivity relationships, we assessed the effects of productivity on diversity metrics including species richness, phylogenetic diversity (*p‐*sesMNTD), and functional diversity (*f‐*sesMNTD; Appendices [Link], [Link]). Prior to selecting a final model, we tested various regression forms including simple linear, second‐degree polynomial (full quadratic), and quadratic‐only models. These models were each applied using ordinary least squares (OLS), 95% quadratic quantile regression, and generalized linear mixed models (GLMMs), following approaches from Fraser et al. (2015), Lisner et al. (2021), and Du et al. (2022). We initially used OLS regression with a second‐degree polynomial to assess whether the relationship was quadratic, i.e., exhibited a hump‐shaped pattern (Lisner et al. 2021).

We further evaluated whether model fit improved using 95% quantile regression, which can better capture upper‐bound relationships in diversity‐productivity relationship patterns (Pierce 2014; Fraser et al. 2015). However, this approach has been criticized for its sensitivity to random variation and assumptions of uniform distribution (Grace et al. 2014). In addition, GLMMs were tested, as they offer a parsimonious and interpretable framework for analyzing count and non‐normally distributed data (Schielzeth 2010). All the candidate models were evaluated using Akaike's Information Criterion (AIC). GLMMs were selected as the best‐supported models based on AIC comparisons; cases where nonlinear models showed marginally lower AIC values were considered equivalent and did not justify additional complexity. We performed stepwise model selection using the *buildglmmTMB* function from the “buildmer” R package (version 2.11; Voeten 2023). Backward stepwise regression was employed due to its effectiveness in handling complex ecological datasets (Adjemian et al. 2006; López‐Ramírez et al. 2024). This approach begins with the full model and iteratively removes non‐significant predictors based on changes in log‐likelihood, continuing until the most parsimonious model is identified.

For the full model assessing site effect (Question 1), we used GLMMs, specifying a generalized Poisson distribution with a log link function when species richness was the response variable, and a Gaussian distribution when either *p‐*sesMNTD or *f‐*sesMNTD was the response variable (Appendix S9). Fixed effects included aboveground live biomass, site, and their interactions. Block nested within year was specified as a random effect to account for repeated measures. The post hoc analysis was performed using the *emtrends* function from the “emmeans” R package (version 1.11.0), but only when the main effect of aboveground live biomass or the site × aboveground live biomass interaction was significant in the optimal model.

For the full model assessing seed source of dominant species and rainfall treatment in each site (Question 2), we used GLMMs, specifying a generalized Poisson distribution with a log link function when species richness was the response variable, and a Gaussian distribution when either *p‐*sesMNTD or *f‐*sesMNTD was the response variable. Fixed effects included aboveground live biomass, seed source, rainfall treatment, and interactions in dry (Hays, KS), mesic (Manhattan, KS), and wettest site (Carbondale, IL). The three‐way interaction among aboveground live biomass, seed source, and rainfall treatment was excluded to avoid overfitting, as higher order interactions lead to convergence problems and inflated standard errors (Myllys et al. 2016; Karimi et al. 2022). Fixed effects at the driest site (Colby, KS) included aboveground live biomass (linear and quadratic terms), seed source, and their interaction, with rainfall treatment excluded due to the absence of rainout treatment. Block nested within year was specified as a random effect to account for repeated measures. The post hoc analysis was performed using the *emtrends* function from the “emmeans” R package (version 1.11.0), but only when the main effect of aboveground live biomass or its interaction with seed source or rainfall treatment was significant.

To address Question 3, we evaluated the ability of diversity estimators, including direct estimators such as aboveground live biomass (Lisner et al. 2021) and indirect estimators such as seed source of dominant species (Mendola et al. 2015; Stahlheber et al. 2020) and rainfall (Rowland et al. 2015; Becknell et al. 2021), to predict diversity metrics using generalized boosted regression trees (BRT). Models were implemented with the *gbm* function in the “gbm3” R package (version 3.0; Ridgeway 2024), which inherently accounts for data nonlinearities and interactions among variables.

We analyzed the influence of each productivity estimator on species richness, phylogenetic diversity (*p‐*sesMNTD), and functional diversity (*f‐*sesMNTD) separately for each of the four sites (Question 3). Rainfall factor was excluded at the driest site (Colby, KS) due to the absence of rainout shelter treatment. We averaged estimator influence from 3000 iterations for models at the dry (Hays, KS), mesic (Manhattan, KS), and wettest (Carbondale, IL) sites, and from 1500 iterations at the driest site (Colby, KS), reflecting the feasible number of plots and parameter stabilization. Species richness models were fitted with a Poisson distribution, while *p‐*sesMNTD and *f‐*sesMNTD models used a Gaussian distribution. All models were run with a bag fraction of 0.8, an interaction depth of 3, and a shrinkage (or learning rate) of 0.01. To prevent overfitting, the optimal number of trees was determined through cross‐validation using the *gbm.perf* function from the “gbm3” R package (version 3.0; Pistón et al. 2019). Aboveground live biomass data were log‐transformed before analysis to correct for highly positive skewness.

## 3 Results

### 3.1 Site Effect

**FIGURE 2:**
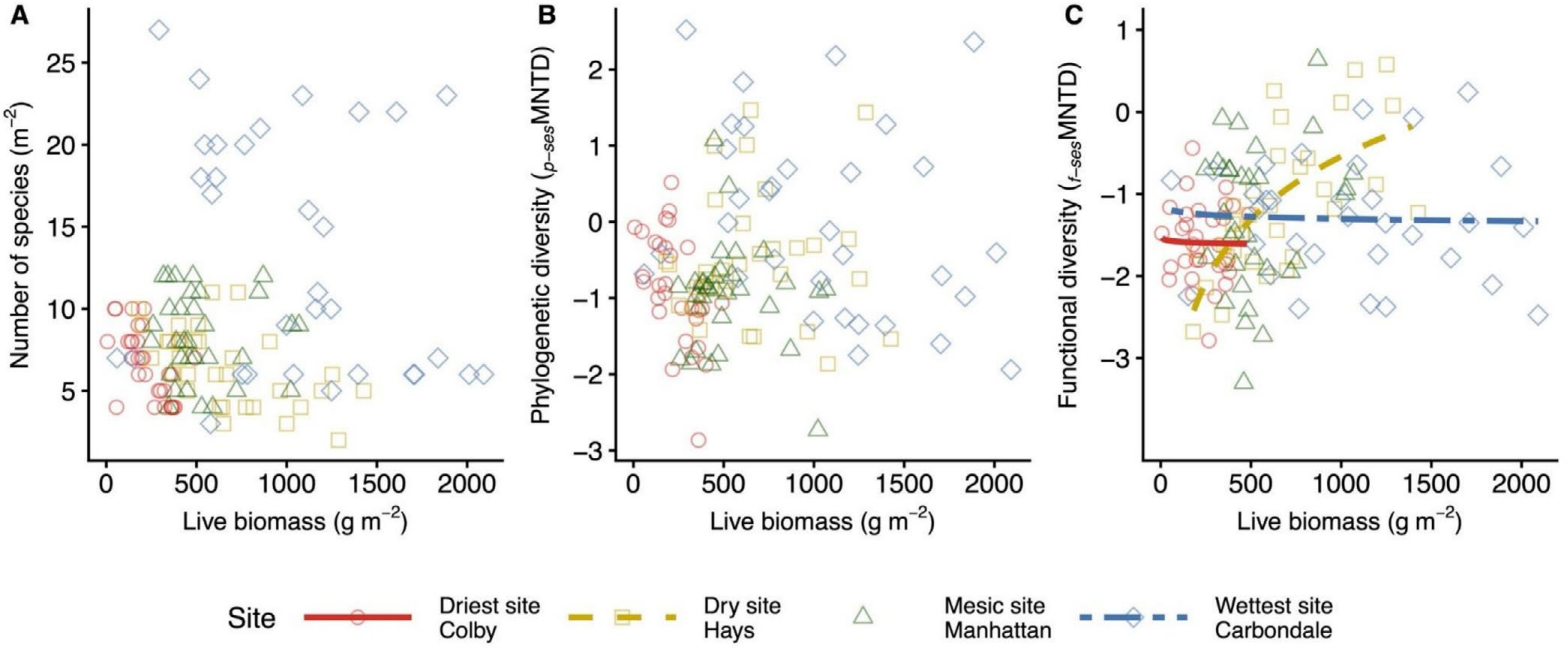
Fitted diversity‐productivity relationships across sites based on (A) species richness (m^−2^), (B) phylogenetic diversity (*p‐*sesMNTD), and (C) functional diversity (*f‐*sesMNTD). Symbols and line types represent sites: Circle with red solid line = driest site (Colby, KS), square with gold dashed line = dry site (Hays, KS), triangle with green dotted line = mesic site (Manhattan, KS), and diamond with blue dot‐dash line = wettest site (Carbondale, IL). Significant relationships are shown with regression lines. ***p < 0.001.

Site effects, representing differences among locations along a regional rainfall gradient in the US Great Plains, were not evident in the species richness‐productivity (Figure 2A) or phylogenetic diversity (*p‐*sesMNTD)‐productivity relationships (Figure 2B; Appendix S9). In contrast, the relationship between functional diversity (*f‐*sesMNTD) and productivity varied across site locations [$\chi^{2}$_(degrees of freedom=3)_ = 19.98, *p* < 0.001; Figure 2C]. Post hoc analysis of the functional diversity‐productivity relationship revealed site‐specific differences in slopes, with the dry site (Hays, KS) showing a strong positive productivity trend compared with both the driest site (Colby, KS; Δslope_[difference in slopes]_ = 1.13 ± 0.27 SE, *p* < 0.001) and the wettest site (Carbondale, IL; Δslope = 1.15 ± 0.28 SE, *p* < 0.001) where there were shallow negative relationships. In general, functional diversity trends at the driest (Colby, KS), mesic (Manhattan, KS), and wettest (Carbondale, IL) sites were non‐significant.

### 3.2 Richness‐Aboveground Live Biomass Patterns

**FIGURE 3:**
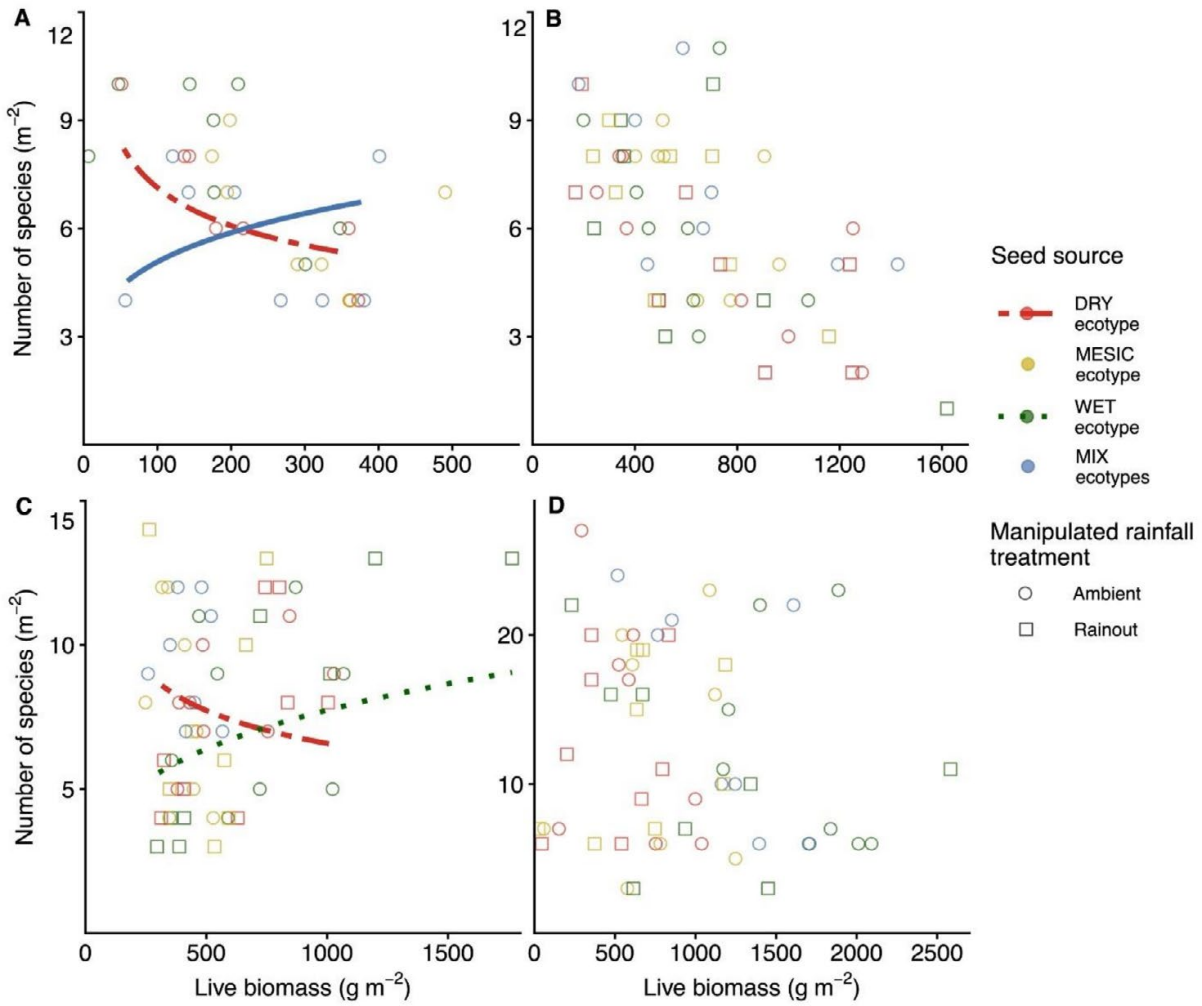
Fitted relationships between species richness (m^−2^) and productivity (g m^−2^) among seed sources representing different ecotypes of dominant species at four sites: (A) driest site (Colby, KS), (B) dry site (Hays, KS), (C) mesic site (Manhattan, KS; inset shows rainfall treatment effects: Solid line = ambient, dotted line = rainout), and (D) wettest site (Carbondale, IL). Symbol colors and line styles indicate ecotypic sources: Red dot‐dash = DRY, gold dashed = MESIC, green dotted = WET, and blue solid = MIX, while symbol shapes denote rainfall treatments (circle = ambient, square = rainout). Regression lines are shown for significant relationships. *p < 0.05; **p < 0.01.

At the driest site (Colby, KS), the relationship between species richness and productivity varied among seed sources [$\chi^{2}$_(3)_ = 10.61, *p* = 0.014; Figure 3A]. *Post hoc* analysis of the richness‐productivity relationship revealed ecotype‐specific differences in slopes, with DRY ecotype plots exhibiting a stronger negative trend, whereas MIX‐source plots showed a positive relationship between richness and productivity (Δslope = −0.45 ± 0.16 SE, *p* = 0.029), and no other pairwise contrasts were significant.

At the dry site (Hays, KS), the richness‐productivity relationship did not differ by seed source or rainfall treatment (Figure 3B), indicating a consistent pattern across ecotypes of dominant species and experimental drought treatments.

At the mesic site (Manhattan, KS), the richness‐productivity relationship varied by seed source [$\chi^{2}$_(3)_ = 13.44, *p* = 0.004] (Figure 3C). Post hoc analysis of the richness‐productivity relationship revealed ecotypic differences in slopes, with DRY ecotype plots exhibiting a stronger negative productivity trend, whereas WET‐ecotype plots showed a positive relationship between richness and productivity (Δslope = −0.51 ± 0.16 SE, *p* = 0.009), and no other pairwise contrasts were significant.

At the wettest site (Carbondale, IL), the richness‐productivity relationship did not vary by seed source or rainfall treatment (Figure 3D), similar to the pattern observed at the dry site (Hays, KS).

### 3.3 Phylogenetic Diversity‐Aboveground Live Biomass Patterns

**FIGURE 4:**
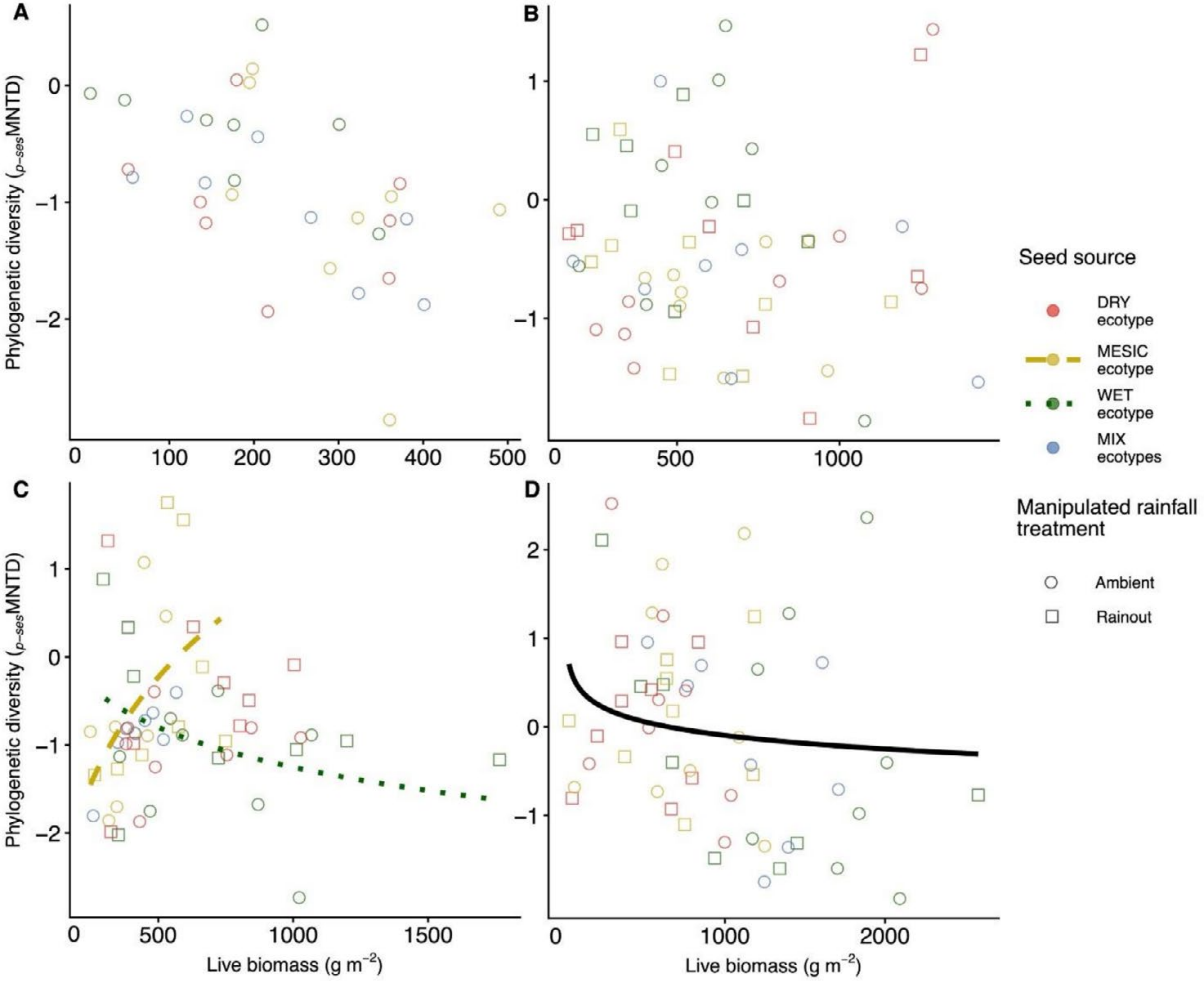
Fitted relationships between phylogenetic diversity (*p‐*sesMNTD) and productivity (g m^−2^) among seed sources representing different ecotypes of dominant species at four sites: (A) driest site (Colby, KS), (B) dry site (Hays, KS), (C) mesic site (Manhattan, KS), and (D) wettest site (Carbondale, IL). Symbol colors and line styles indicate ecotypic sources: Red dot‐dash = DRY, gold dashed = MESIC, green dotted = WET, and blue solid = MIX, while symbol shapes denote rainfall treatments (circle = ambient, square = rainout). Regression lines are shown for significant relationships. *p < 0.05; **p < 0.01.

At the driest site (Colby, KS), the *p‐*sesMNTD‐productivity relationship was consistent across seed sources (Figure 4A). Similarly, at the dry site (Hays, KS), the relationship did not differ by seed source or rainfall treatment (Figure 4B). In contrast, at the mesic site (Manhattan, KS), the strength of the relationship between *p‐*sesMNTD and productivity varied by seed source [$\chi^{2}$_(3)_ = 12.33, *p* = 0.006; Figure 4C]. Post hoc analysis of the phylogenetic diversity‐productivity relationship indicated ecotype‐specific differences in slopes, with plots planted with the local MESIC‐ecotype exhibiting a strong positive productivity trend, whereas plots planted with the WET‐ecotype showed a negative trend (Δslope = 2.40 ± 0.73 SE, *p* = 0.011), indicating that local and non‐local seed sources differed in their phylogenetic community responses to productivity. At the wettest site (Carbondale, IL), *p‐*sesMNTD decreased with productivity [$\chi^{2}$_(1)_ = 4.23, *p* = 0.040; Figure 4D], with no evidence of variation by seed source or rainfall treatment.

### 3.4 Functional Diversity‐Aboveground Live Biomass Patterns

**FIGURE 5:**
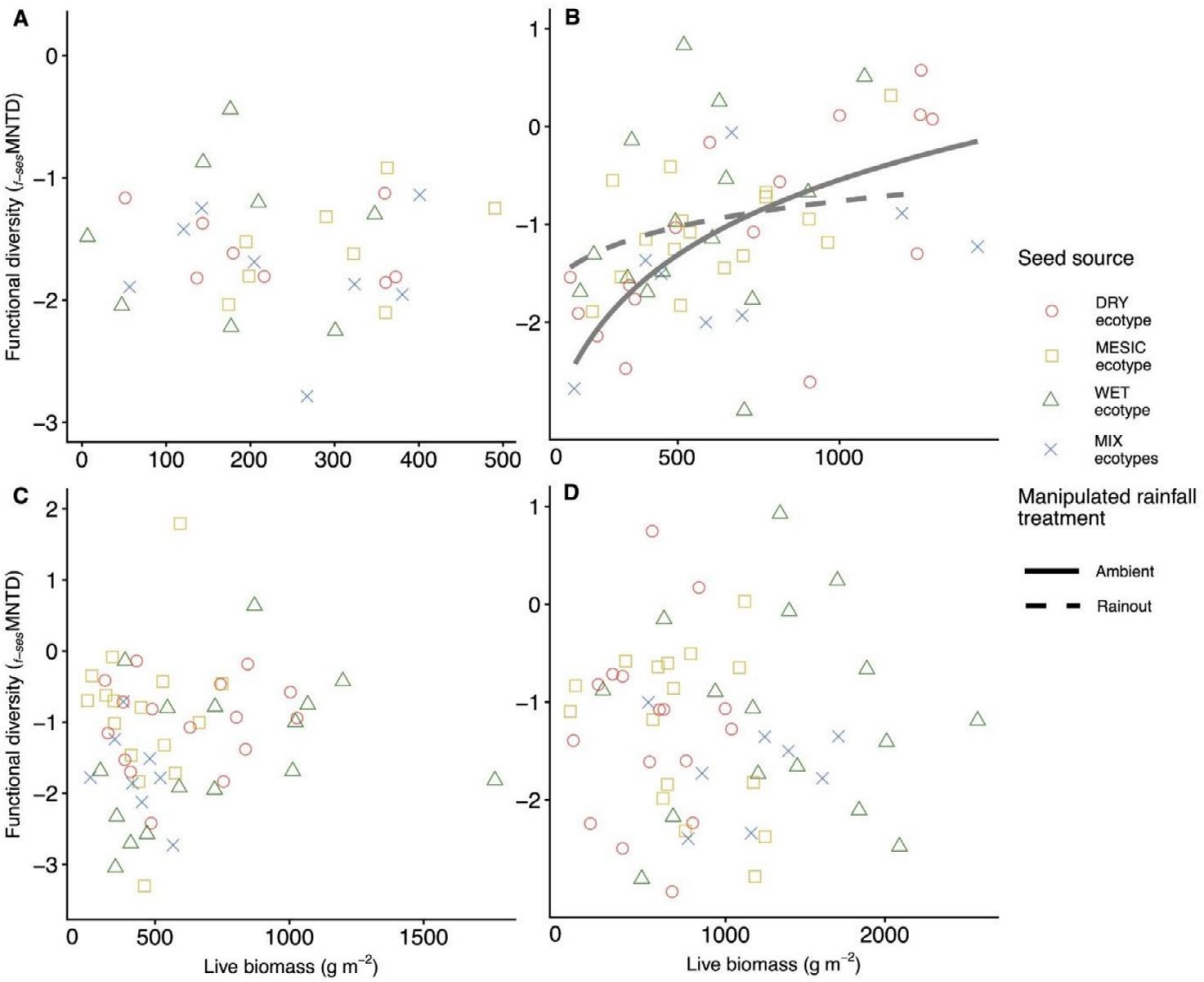
Fitted relationships between functional diversity (*f‐*sesMNTD) and productivity (g m^−2^) among seed sources representing different ecotypes of dominant species at four sites: (A) driest site (Colby, KS), (B) dry site (Hays, KS), (C) mesic site (Manhattan, KS), and (D) wettest site (Carbondale, IL). Line styles indicate rainfall treatments (circle = ambient, square = rainout), while symbol shapes and colors denote ecotypic sources: Red dot‐dash = DRY, gold dashed = MESIC, green dotted = WET, and blue solid = MIX. Regression lines are shown for significant relationships. **p < 0.01.

At the driest (Colby, KS), mesic (Manhattan, KS), and wettest (Carbondale, IL) sites, no relationships were observed between *f‐*sesMNTD and productivity across seed sources and rainfall treatments (Figure 5A,C,D). In contrast, at the dry site (Hays, KS), the strength of the positive functional diversity‐productivity relationship varied by rainfall treatment [$\chi^{2}$_(1)_ = 4.22, *p* = 0.040] (Figure 5B). Post hoc analysis revealed a stronger positive slope under ambient conditions (slope = 1.11 ± 0.24 SE) compared to rainout (slope = 0.38 ± 0.26 SE).

### 3.5 Differential Explanatory Power of Diversity Estimators

**FIGURE 6:**
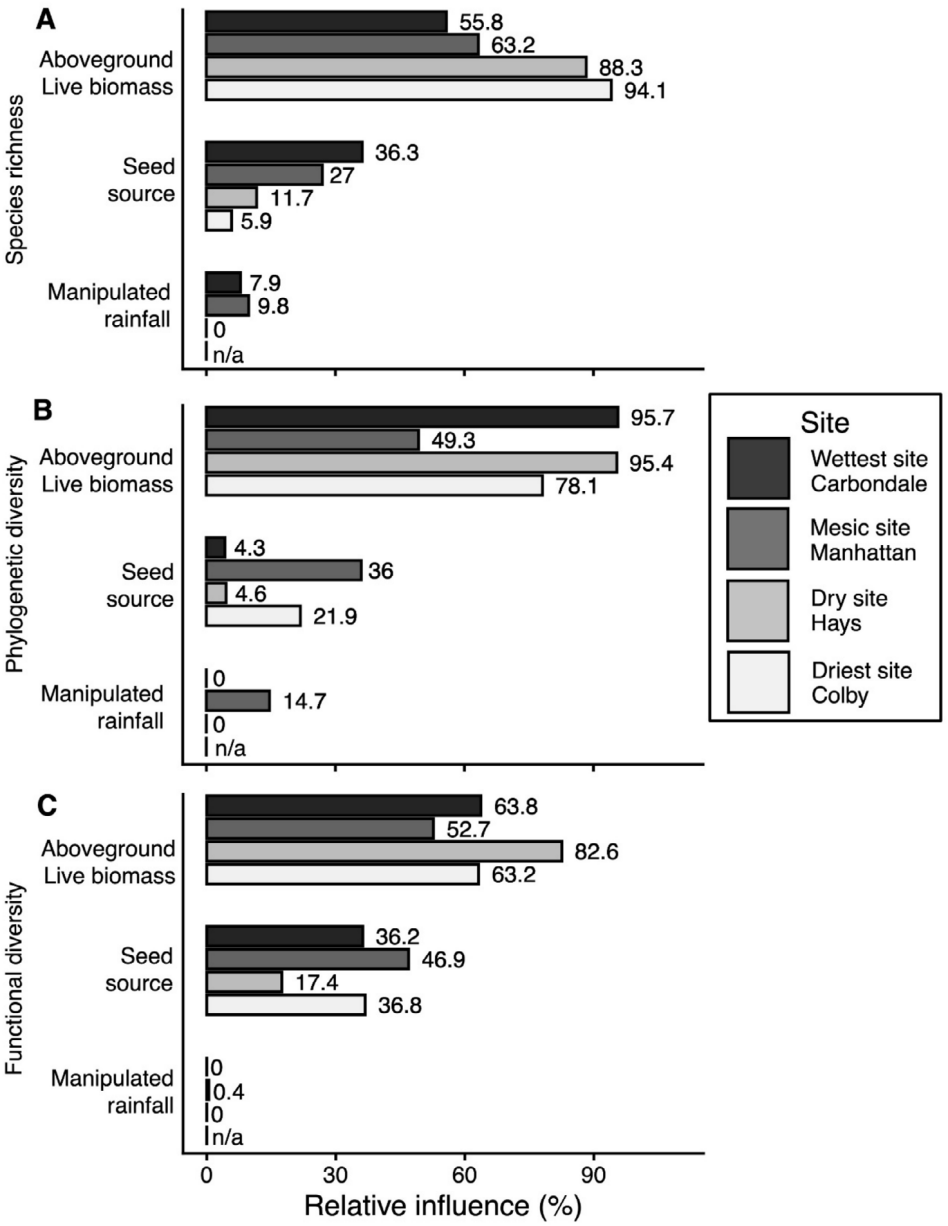
(A) Species richness (m^−2^), (B) phylogenetic diversity (*p‐*sesMNTD), and (C) functional diversity (*f‐*sesMNTD) were analyzed to determine how strongly they were influenced by aboveground live biomass, dominant species source, and manipulated rainfall using generalized boosted regression trees. The numbers at the end of each bar show the relative influence (%) of each explanatory factor. “n/a” denotes that no manipulated rainfall treatment was implemented at the driest site (Colby, KS).

Aboveground live biomass was the dominant predictor of species richness across all sites (Figure 6A; Appendix S10). Its explanatory power was highest at the driest site (94%) and declined progressively toward wetter sites, accounting for 88% at the dry site, 63% at the mesic site, and 56% at the wettest site. Seed source had a secondary but increasing influence along the rainfall gradient, contributing 6% at the driest site, 12% at the dry site, 27% at the mesic site, and 36% at the wettest site. In contrast, manipulated rainfall had minimal explanatory power, contributing modestly at the mesic (10%) and wettest (8%) sites and having negligible or no effect at the remaining sites.

Aboveground live biomass was the primary driver of phylogenetic diversity across most sites (Figure 6B; Appendix S10). Its explanatory power was highest at the wettest (96%) and dry (95%) sites, remained strong at the driest site (78%), and declined markedly at the mesic site (49%). In contrast, seed source exerted a substantial influence primarily at the mesic site (36%) and, to a lesser extent, at the driest site (22%), but had minimal effects at the wettest (4%) and dry (5%) sites. Manipulated rainfall contributed moderately to phylogenetic diversity at the mesic site (15%) and was negligible or not applicable at the remaining sites.

In contrast to phylogenetic diversity, functional diversity showed a weaker overall dependence on aboveground live biomass across sites (Figure 6C; Appendix S10). Although aboveground live biomass remained the primary driver, its explanatory power was consistently lower than that observed for phylogenetic diversity, peaking at the dry site (83%) and declining to 64% at the wettest site, 63% at the driest site, and 53% at the mesic site. Correspondingly, seed source played a more prominent role in shaping functional diversity, contributing substantially at the mesic site (47%) and remaining moderate at the wettest (36%) and driest (37%) sites, before declining at the dry site (17%). Manipulated rainfall showed no detectable contribution to functional diversity at any site.

## 4 Discussion

### 4.1 Site Effect

In evaluating whether the diversity‐productivity relationship varies across the rainfall gradient (Question 1), we found site effects only in the functional diversity‐productivity relationship, and our predicted fully unimodal diversity‐productivity pattern was not supported across sites. In fact, recent studies increasingly support the idea that functional diversity‐productivity relationships may become more positive in water‐limited environments (Aoyama et al. 2023). At the dry site, functional diversity increased as productivity rose, suggesting that higher productivity supported a broader range of functional strategies. This positive link between functional diversity and productivity in dry systems has also been observed in long‐term dryland (Hisano et al. 2024). In contrast, non‐significant linear patterns at the driest (Cobly, KS) and wettest (Carbondale, IL) sites suggest that trait‐mediated coexistence may be reduced or altered, possibly due to factors including aridity filter at the driest site (Kim and Ohr 2020) or competitive exclusion by dominant species at the wettest site (Shovon et al. 2022). These findings highlight the context‐dependent influence of functional traits on diversity‐productivity relationships and emphasize the need to consider local climate, soil, and vegetation history in guiding restoration strategies. Site effects were not detected in the relationships involving species richness or phylogenetic diversity, showing that ecosystem functions such as productivity are primarily driven by the functional traits of dominant species rather than species number or evolutionary history (Liu et al. 2015; van der Sande et al. 2018; Avolio et al. 2019; Engel et al. 2023). Phylogenetic diversity reflects trait variation and co‐evolutionary history (Flynn et al. 2011). Functional diversity, however, more directly captures the mechanisms underlying ecosystem processes, such as niche complementarity and selection effects (van Zuidam et al. 2019), and clarifies the impacts of species loss (Flynn et al. 2011) compared with species richness and phylogenetic diversity.

### 4.2 Ecotypic Effect in Each Site

In our analysis of how ecotypes of dominant species influence diversity‐productivity relationships (Question 2), ecotype effects were site‐contingent along the rainfall gradient, and contrary to our prediction, home‐site advantage associated with ecotype nativeness was detected. Recent work across precipitation gradients similarly shows that water availability regulates the balance between abiotic filtering and biotic interactions, thereby shaping diversity‐productivity relationships through shifts in stress tolerance and competition (Jiang et al. 2024; Hisano et al. 2024). At the driest site, the species richness‐productivity relationship diverged strongly between DRY‐ and MIX‐ecotypes, suggesting that community assembly shifted under intense water limitation. At both the driest and mesic sites, the negative relationship observed in the DRY ecotype is consistent with theory predicting that aridity filtering increases in stressful environments, allowing drought‐adapted dominants to monopolize resources and reduce coexistence (Grime 1973; Adler et al. 2011). Comparable dominance‐driven biodiversity declines under environmental stress have recently been documented in experimental grasslands, where environmental perturbations favored stress‐tolerant taxa and reduced opportunities for coexistence (Aoyama et al. 2023).

MIX‐ecotype plots showed a positive richness‐productivity trend, possibly driven by niche differentiation among ecotypic strategies (Cardinale et al. 2006), a pattern consistent with the stress‐gradient hypothesis that emphasizes stronger facilitative or cooperative interactions under climatic stress. Together, these contrasting patterns suggest that ecotypic mixing may help maintain diversity under extreme aridity by reducing dominance‐driven exclusion observed in single‐source DRY populations.

At the mesic site (Manhattan, KS), productivity gains in WET‐ecotype plots corresponded with rising richness but reduced phylogenetic diversity, implying that WET‐ecotype dominance under intermediate rainfall may relax competitive exclusion primarily for evolutionarily similar taxa. Similar shifts toward competitive structuring under moderate precipitation regimes have been reported along rainfall gradients, where increasing moisture reduces environmental filtering and favors coexistence among functionally similar species (Jiang et al. 2024). Conversely, productivity gains in local MESIC‐ecotype plots corresponded with greater phylogenetic diversity, suggesting that locally adapted dominants may generate fine‐scale light and moisture heterogeneity that supports the recruitment of distantly related lineages. This mechanism aligns with studies showing that environmental heterogeneity created by dominant species can expand niche space and stabilize productivity across phylogenetically diverse assemblages (Hisano et al. 2024).

At the wettest site, phylogenetic diversity declined toward a neutral baseline (around 0) as productivity increased, suggesting that community assembly became more stochastic under favorable climatic conditions with reduced environmental filtering. Recent synthesis work indicates that high resource availability can weaken deterministic assembly processes and promote more neutral or stochastic phylogenetic structure as competitive hierarchies relax (Li et al. 2025). In contrast, functional diversity‐productivity relationships did not vary among ecotypes across sites, indicating that multi‐trait functional metrics were comparatively less responsive to seed source differences.

At the dry site (Hays, KS), rainfall manipulation coincided with peak productivity driven by arid‐adapted species such as kochia ( *Bassia scoparia* ) and clammy groundcherry ( *Physalis heterophylla* ), which exhibit larger leaf area but lower height and leaf nitrogen content. Trait combinations associated with drought tolerance may promote productivity without necessarily increasing functional diversity, reflecting convergence toward stress‐adapted strategies under strong environmental filtering (Laughlin 2014; Gross et al. 2017). No ecotype effects on any diversity‐productivity relationships were detected at the dry (Hays, KS) or wettest (Carbondale, IL) sites, possibly due to resource saturation or abiotic filtering (e.g., experimental drought effect), which may override trait differences among ecotypes and lead to convergent community responses regardless of seed origin. Similar patterns have been reported in restoration and drought‐manipulation experiments where environmental constraints dominated assembly outcomes and reduced the influence of intraspecific variation on community structure (Helsen et al. 2017; Funk et al. 2024).

### 4.3 Relative Contributions of Diversity Estimators

To evaluate the relative contributions of diversity estimators (Question 3), we found that aboveground live biomass played a central role in shaping community diversity across the rainfall gradient (Grace 2001). Aboveground live biomass consistently emerged as the strongest predictor of species richness (Lisner et al. 2021), as well as phylogenetic and functional diversity across all sites, with its explanatory power peaking at the driest site (Cadotte et al. 2009; Thompson et al. 2015). In contrast, the effects of seed source and manipulated rainfall were more site‐specific, with their influence varying depending on local climate conditions (Zobel and Pärtel 2008; Schöb et al. 2015; Wagg et al. 2017; Kreyling et al. 2017; Cheng et al. 2021). Seed sources moderately affected species richness at the wettest site and both phylogenetic and functional diversity at the mesic site, suggesting that locally adapted dominant species may influence community structure by outcompeting subordinate species in wetter regions under favorable or moderate climatic conditions. Manipulated rainfall had limited overall influence but contributed modestly at the mesic and wettest sites, indicating that experimental drought during the growing season has a relatively weak influence on diversity‐productivity relationships compared to the stronger effects of aboveground live biomass (Yan et al. 2015). Together, these findings suggest that aboveground live biomass remains the dominant driver of diversity patterns, as high‐biomass intensifies competition for light, water, and nutrients, influencing species coexistence and exclusion (Craine and Dybzinski 2013). However, the ecotypic identity of dominant species and experimental rainfall treatments can act as important secondary factors, depending on site‐specific conditions and the diversity metrics considered.

By examining grassland communities along a precipitation gradient and incorporating both dominant species ecotypes and rainfall treatment, we assessed biodiversity‐productivity relationships at a regional scale. This approach, however, involved several limitations. First, our analysis focused solely on vascular plants, excluding bryophytes and pteridophytes, which can be important components of vegetation in certain grasslands such as dunes, grazed pastures, or mixed shrub savannas (da Silveira et al. 2022). Second, functional diversity was derived from ten traits across more than 90 species. These traits represent key ecological functions, including resource acquisition, reproduction, and dispersal. Nevertheless, they may not capture the full spectrum of functional trade‐offs relevant across the productivity gradient. In addition, while ecotypic variation is known to affect productivity (Fetcher and Shaver 1990; Mendola et al. 2015; Stahlheber et al. 2020), estimates may be less reliable in plots with extensive bare ground such as at the dry and driest sites. Finally, our approach enables simultaneous evaluation of multiple diversity dimensions, narrowing potential mechanisms such as plant‐microbe interactions, though its empirical nature limits causal interpretation.

## 5 Conclusion

Our results demonstrate the relative importance of local and regional processes affecting the role of ecotypes of dominant species controlling the diversity of non‐dominant species (Ricklefs 1987; Whitham et al. 2020). As with many ecological phenomena, the relationship between biodiversity and productivity is mechanistically context‐dependent (Catford et al. 2022) based upon local conditions, and, as we show, the ecotype source of dominant species and the metric of diversity considered. Appreciating the context dependency of ecological phenomena, including diversity‐productivity relationships (Mori et al. 2021) and the effects of species interactions (Chamberlain et al. 2014), has relevance for selecting sources for restorations and needs to be incorporated in management decisions (Klopf et al. 2014; Baer et al. 2019) in light of ongoing increased drought effects in grasslands (Gibson and Newman 2019).

## Author Contributions

**Zhe Ren:** conceptualization (lead), data curation (equal), formal analysis (equal), investigation (equal), methodology (lead), validation (equal), visualization (lead), writing – original draft (lead), writing – review and editing (lead). **David J. Gibson:** conceptualization (equal), data curation (equal), formal analysis (equal), funding acquisition (lead), investigation (equal), methodology (equal), supervision (lead), validation (lead), writing – original draft (equal), writing – review and editing (equal). **David F. Barfknecht:** data curation (equal), formal analysis (supporting), funding acquisition (equal), writing – original draft (equal), writing – review and editing (equal). **Sara G. Baer:** investigation (equal), project administration (equal), writing – original draft (equal), writing – review and editing (equal). **Matthew B. Galliart:** data curation (equal), resources (equal), writing – original draft (supporting), writing – review and editing (supporting). **Jack R. Sytsma:** data curation (equal), investigation (supporting), project administration (supporting), resources (supporting), validation (equal), writing – original draft (equal), writing – review and editing (equal). **Loretta C. Johnson:** conceptualization (equal), funding acquisition (equal), investigation (equal), project administration (lead), supervision (lead), writing – original draft (equal), writing – review and editing (equal).

## Funding

This work was supported by Division of Undergraduate Education, 1758497, 1949969; National Institute of Food and Agriculture, 2008‐35100‐04545.

## Conflicts of Interest

The authors declare no conflicts of interest.

## Supporting information


**Appendix S1:** Plant species and seeding density used for common garden establishment (Johnson et al. 2015).
**Appendix S2:** Trait measurements (mean ± SE) of ecotypes (WET, MESIC, or DRY) of each dominant grass species ( *Andropogon gerardi* or *Sorghastrum nutans* ). Sample sizes (*n*) refer to the number of individual plants from which traits were measured (mean ± SE) followed by identical letters were not significantly different from each other (experiment‐wide *α* = 0.05, Tukey adjusted; Ren et al. 2024). Representative photographs of WET and DRY ecotypes of *A. gerardi* (MESIC‐ecotype absent).
**Appendix S3:** Maximum likelihood phylogenies for taxa recorded in (A) 2012 and (B) 2021 surveys across all sites.
**Appendix S4:** Functional trait dendrograms for taxa recorded in (A) 2012 and (B) 2021 surveys across all sites.
**Appendix S5:** Correlation among indices of diversity. Blue and red colors reflect levels of significant correlation (*p* < 0.05) indicated along the bottom bar; × indicates insignificant correlations (*p* > 0.05). F0, F1, F2, Hill's numbers of functional trait diversity (Chao et al. 2014); fmntd, functional mean taxon distance; fmpd, functional mean pairwise distance; fnri, functional net relatedness index; fnti, functional nearest taxon index; P0, P1, P2, Hill's numbers of phylogenetic diversity; pmntd, phylogenetic mean taxon distance; pmpd, phylogenetic mean pairwise distance; pnri, phylogenetic net relatedness index; pnti, phylogenetic nearest taxon index; Prod_no_litter, live plant biomass; T0, T1, T2, Hill's numbers of taxonomic diversity.

**Appendix S6:** Plot‐level data on productivity and multidimensional diversity metrics.

**Appendix S7:** R scripts used for data processing and statistical analysis.

**Appendix S8:** Species abundance and functional trait data.

**Appendix S9:** Site‐level summary of biomass and plant diversity (mean ± SE) along a rainfall gradient.
**Appendix S10:** Site‐level cross‐validated root mean square error (RMSE) for biodiversity metrics along a rainfall gradient, reported to quantify the predictive accuracy of the boosted regression tree models.

## Data Availability

The data and codes used in this study are available with the corresponding Supporting Information.
