# Supplementary material for: Ecotypic Identity and Manipulated Rainfall Modulate Diversity‐Productivity Relationships Across a Precipitation Gradient
Source: Ecol Evol. 2026 Apr 9;16(4):e73464. doi: 10.1002/ece3.73464 (PMC13065499; doi:10.1002/ece3.73464)

**SUPPLEMENTAL MATERIALS S1-S5**

**Ecotypic identity and manipulated rainfall modulate diversity-productivity relationships across a precipitation gradient**

**Authorship**

Zhe Ren^1, 2, 3^, David J. Gibson^3^, David F. Barfknecht^3, 4^, Sara G. Baer^5^, Matthew B. Galliart^6^, Jack R. Sytsma^7^, Loretta C. Johnson^7^

^1^ Department of Biological and Clinical Sciences, University of Central Missouri, Warrensburg, Missouri, USA

^2^ Department of Botany and Plant Pathology, Purdue University, West Lafayette, Indiana, USA

^3^ School of Biological Sciences, Southern Illinois University, Carbondale, Illinois, USA

^4^ Department of Forest and Wildlife Ecology, University of Wisconsin, Madison, Wisconsin, USA

^5^ Department of Ecology & Evolutionary Biology and Kansas Biological Survey & Center for Ecological Research, University of Kansas, Lawrence, Kansas, USA

^6^ Department of Biological Sciences, Fort Hays State University, Hays, Kansas, USA

^7^ Division of Biology, Kansas State University, Manhattan, Kansas, USA

**Correspondence**

Zhe Ren, Purdue University, 1370 Lilly Hall of Life Sciences, 915 Mitch Daniels Blvd, West Lafayette, Indiana, USA, 47907

Email address: ren256@purdue.edu

**Appendix S1 Plant species and seeding density used for common garden establishment (Johnson et al., 2015)**

|  | **Planted species** | **Family** | **Source** | **Seeding rate (seeds m^-2^)** |
| --- | --- | --- | --- | --- |
| **Grasses** | *Andropogon gerardi* | Poaceae | Local^1^ | 270 |
|  | *Sorghastrum nutans* | Poaceae | Local^2^ | 70 |
|  | *Elymus canadensis* | Poaceae | Commercial^3^ | 30 |
| **Forbs** | *Asclepias tuberosa* | Apocynaceae | Commercial^3^ | 30 |
|  | *Chamaecrista fasciculata* | Fabaceae | Commercial^3^ | 30 |
|  | *Dalea purpurea* | Fabaceae | Commercial^3, 4^ | 30 |
|  | *Monarda fistulosa* | Lamiaceae | Commercial^3^ | 30 |
|  | *Solidago rigida* | Asteraceae | Commercial^3^ | 30 |
|  | *Penstemon digitalis* | Plantaginaceae | Commercial^3^ | 30 |
|  | *Ruellia humilis* | Acanthaceae | Commercial^3^ | 30 |
|  | Total seeds (m^-2^) |  |  | 580 |

^1^Indicating seeds were collected from multiple remnants; ^2^Indicating seeds were collected from one remnant prairie within the native habitat for each ecotype (e.g., DRY ecotype in Hays, KS, US, 38°51′13.2′′N, 99°19′08.6′′W; MESIC ecotype in Manhattan, KS, US, 39°08′22.3′′N, 96°38′23.3′′W; and WET ecotype in Carbondale, IL, US, 37°41′47.0′′N, 89°14′19.2′′W); ^3^Indicating seeds purchased from Ion Exchange, Inc., Harpers Ferry, IA, US; ^4^*Dalea purpurea* was initially sown for the experiment but was absent in field surveys from 2012 to 2019.

**Appendix S2 Trait measurements (mean ± standard error) of ecotypes (WET, MESIC, or DRY) of each dominant grass species (*Andropogon gerardi* or *Sorghastrum nutans*). Sample sizes (n) refer to the number of individual plants from which traits were measured. (mean ± standard error) followed by identical letters were not significantly different from each other (experiment-wide α = 0.05, Tukey adjusted; Ren et al., 2024). Representative photographs of WET and DRY ecotypes of *A. gerardi* (MESIC ecotype absent)**

| Functional trait | *Andropogon gerardi*  (n = 10 per ecotype) | | | *Sorghastrum nutans*  (n = 10 per ecotype) | | |
| --- | --- | --- | --- | --- | --- | --- |
|  | ***WET*** | ***MESIC*** | ***DRY*** | ***WET*** | ***MESIC*** | ***DRY*** |
| Height (cm) | 286.7 ± 2.8 **a** | 217.4 ± 3.23 **b** | 201.3 ± 4.47 **c** | 257.0 ± 7.23 **a** | 206.9 ± 5.57 **b** | 208.5 ± 4.02 **b** |
| Leaf Area (cm^2^) | 37.1 ± 1.28 **a** | 27.9 ± 1.38 **b** | 35.7 ± 2.49 **a** | 30.3 ± 2.17 **a** | 28.8 ± 2.24 **a** | 28.8 ± 2.27 **a** |
| Seed Mass (mg) | 3.1 ± 0.06 **a** | 2.7 ± 0.11 **b** | 2.8 ± 0.06 **b** | 2.0 ± 0.05 **b** | 2.4 ± 0.04 **a** | 1.9 ± 0.03 **b** |
| Specific Leaf Area (cm^2^ g^-1^) | 170.0 ± 6.62 **a** | 149.2 ± 20.43 **a** | 210.1 ± 21.95 **a** | 142.7 ± 12.61 **a** | 149.2 ± 21.13 **a** | 137.6 ± 6.92 **a** |
| Leaf N Content (mg g^-1^) | 11.2 ± 0.56 **a** | 10.6 ± 0.78 **a** | 11.7 ± 0.78 **a** | 11.4 ± 0.54 **a** | 10.4 ± 0.56 **a** | 7.2 ± 0.53 **b** |

**
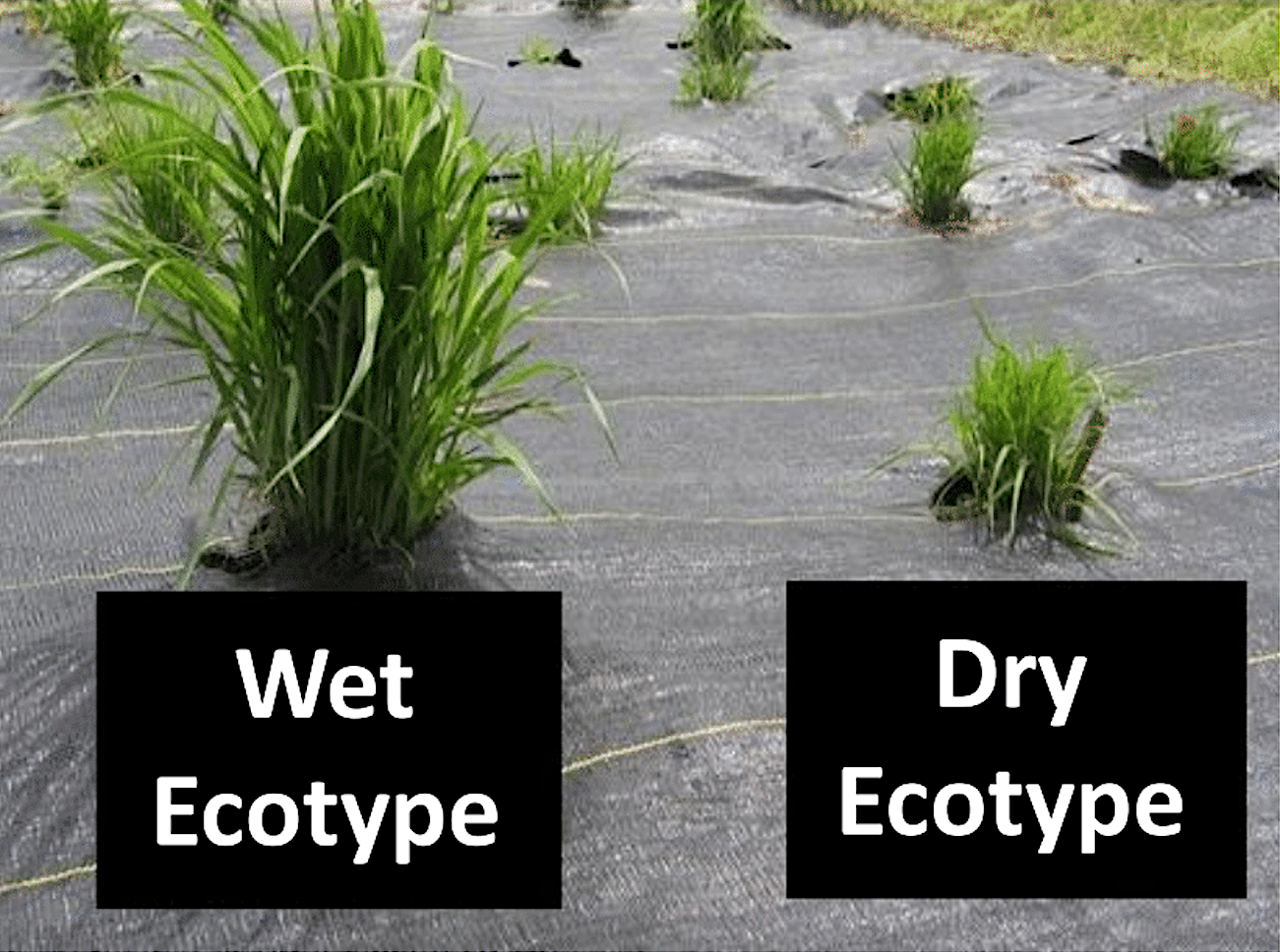
**

**Appendix S3 Maximum likelihood phylogenies for taxa recorded in A) 2012 and B) 2021 surveys across all sites**

A)


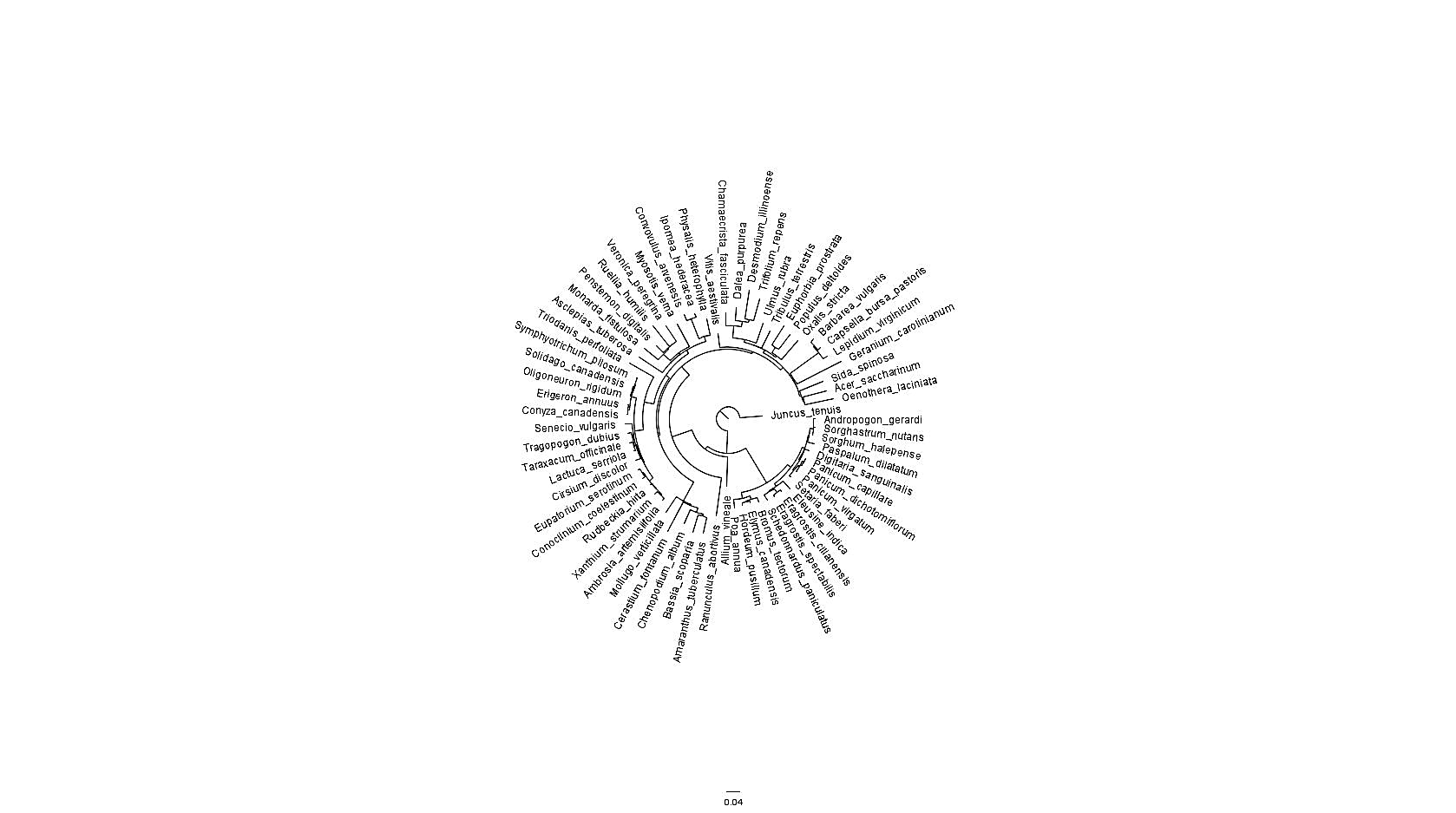


B)

**
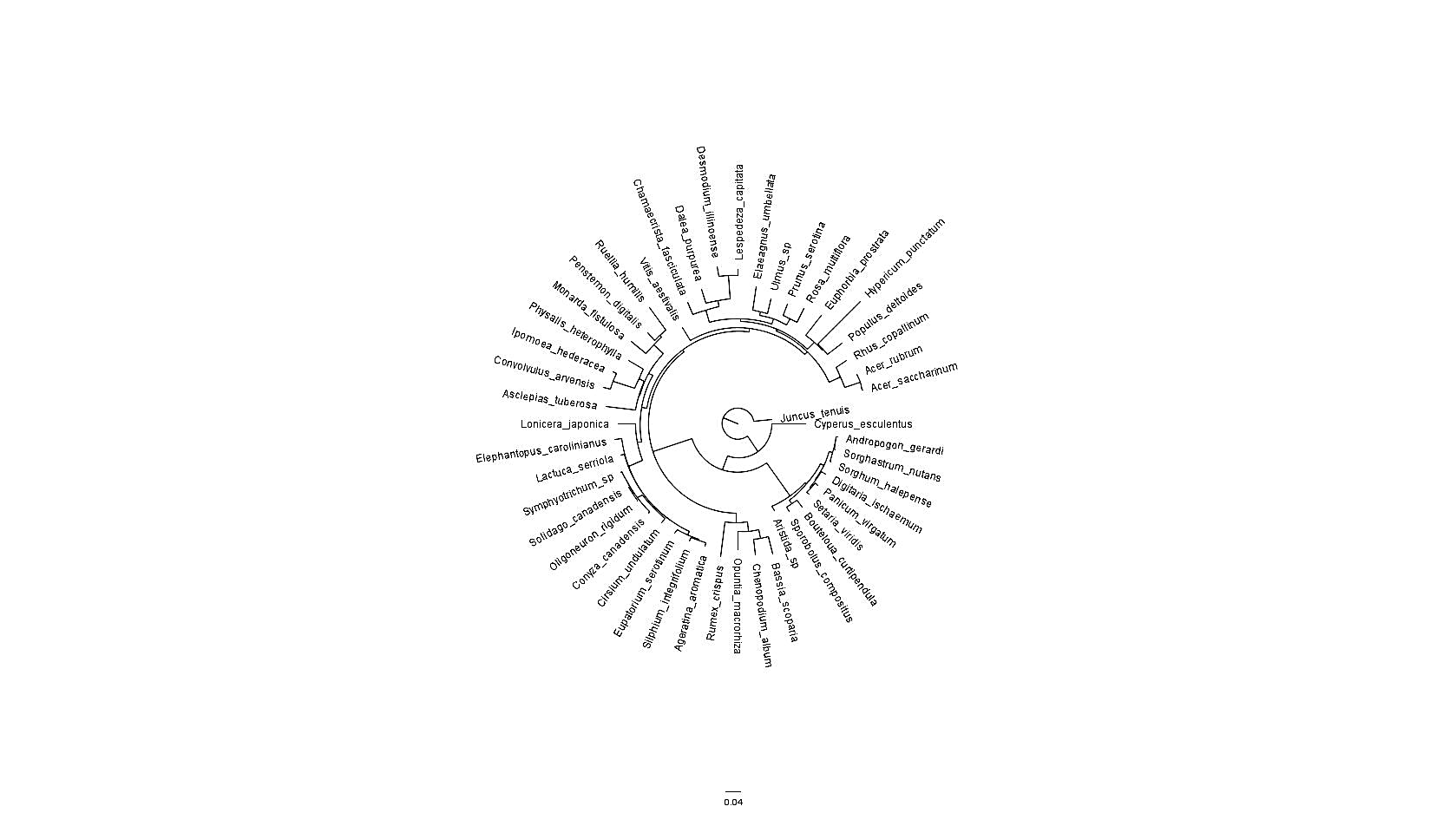
**

**Appendix S4 Functional trait dendrograms for taxa recorded in A) 2012 and B) 2021 surveys across all sites**


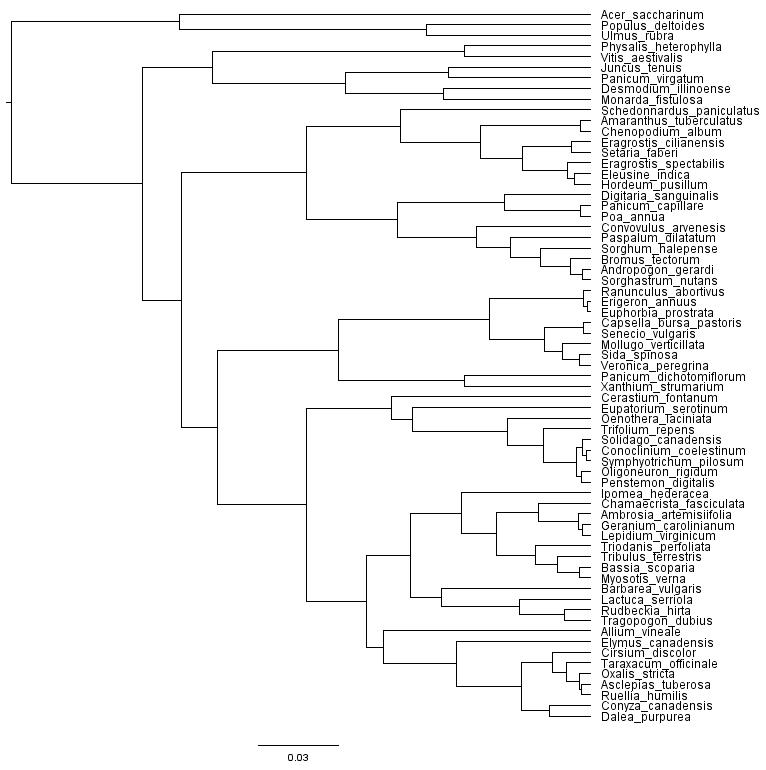


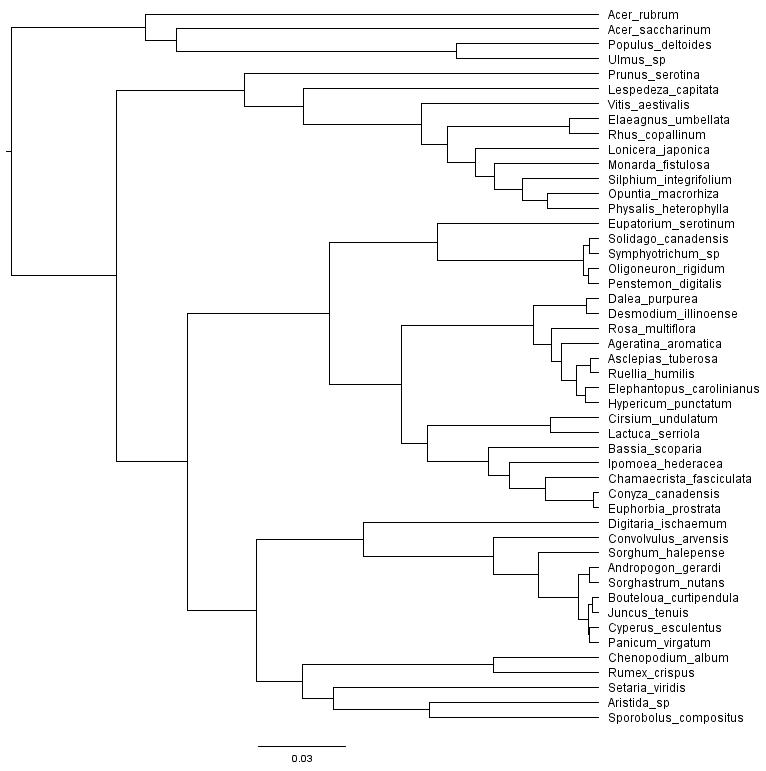


**Appendix S5 Correlation among indices of diversity. Blue and red colors reflect levels of significant correlation (P < 0.05) indicated along the bottom bar;** $\boldsymbol{\times}$ **indicates insignificant correlations (P > 0.05). Prod_no_litter = live plant biomass. T0, T1, T2 = Hill’s numbers of taxonomic diversity, P0, P1, P2 = Hill’s numbers of phylogenetic diversity, F0,F1, F2 = Hill’s numbers of functional trait diversity (Chao et al. 2014), pmpd = phylogenetic mean pairwise distance, pnri = phylogenetic net relatedness index, pmntd = phylogenetic mean taxon distance, pnti = phylogenetic nearest taxon index, fmpd = functional mean pairwise distance, fnri = functional net relatedness index, fmntd = functional mean taxon distance, fnti = functional nearest taxon index.**


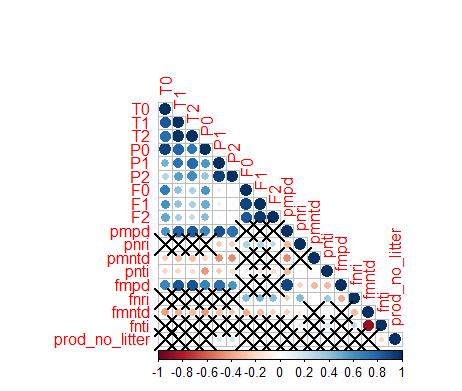

Supplement: Supplementary file 1 — Appendix S1: Plant species and seeding density used for common garden establishment (Johnson et al. 2015). Appendix S2: Trait measurements (mean ± SE) of ecotypes (WET, MESIC, or DRY) of each dominant grass species ( Andropogon gerardi or Sorghastrum nutans ). Sample sizes (n) refer to the number of individual plants from which traits were measured (mean ± SE) followed by identical letters were not significantly different from each other (experiment‐wide α = 0.05, Tukey adjusted; Ren et al. 2024). Representative photographs of WET and DRY ecotypes of A. gerardi (MESIC‐ecotype absent). Appendix S3: Maximum likelihood phylogenies for taxa recorded in (A) 2012 and (B) 2021 surveys across all sites. Appendix S4: Functional trait dendrograms for taxa recorded in (A) 2012 and (B) 2021 surveys across all sites. Appendix S5: Correlation among indices of diversity. Blue and red colors reflect levels of significant correlation (p < 0.05) indicated along the bottom bar; × indicates insignificant correlations (p > 0.05). F0, F1, F2, Hill's numbers of functional trait diversity (Chao et al. 2014); fmntd, functional mean taxon distance; fmpd, functional mean pairwise distance; fnri, functional net relatedness index; fnti, functional nearest taxon index; P0, P1, P2, Hill's numbers of phylogenetic diversity; pmntd, phylogenetic mean taxon distance; pmpd, phylogenetic mean pairwise distance; pnri, phylogenetic net relatedness index; pnti, phylogenetic nearest taxon index; Prod_no_litter, live plant biomass; T0, T1, T2, Hill's numbers of taxonomic diversity. [file ECE3-16-e73464-s004.docx]
