# Supplementary material for: Ecotypic Identity and Manipulated Rainfall Modulate Diversity‐Productivity Relationships Across a Precipitation Gradient
Source: Ecol Evol. 2026 Apr 9;16(4):e73464. doi: 10.1002/ece3.73464 (PMC13065499; doi:10.1002/ece3.73464)
Supplement: Supplementary file 5 — Appendix S9: Site‐level summary of biomass and plant diversity (mean ± SE) along a rainfall gradient. Appendix S10: Site‐level cross‐validated root mean square error (RMSE) for biodiversity metrics along a rainfall gradient, reported to quantify the predictive accuracy of the boosted regression tree models. [file ECE3-16-e73464-s001.docx]

**SUPPLEMENTAL MATERIALS S9-S10**

**Ecotypic identity and manipulated rainfall modulate diversity-productivity relationships across a precipitation gradient**

**Authorship**

Zhe Ren^1, 2, 3^, David J. Gibson^3^, David F. Barfknecht^3, 4^, Sara G. Baer^5^, Matthew B. Galliart^6^, Jack R. Sytsma^7^, Loretta C. Johnson^7^

^1^ Department of Biological and Clinical Sciences, University of Central Missouri, Warrensburg, Missouri, USA

^2^ Department of Botany and Plant Pathology, Purdue University, West Lafayette, Indiana, USA

^3^ School of Biological Sciences, Southern Illinois University, Carbondale, Illinois, USA

^4^ Department of Forest and Wildlife Ecology, University of Wisconsin, Madison, Wisconsin, USA

^5^ Department of Ecology & Evolutionary Biology and Kansas Biological Survey & Center for Ecological Research, University of Kansas, Lawrence, Kansas, USA

^6^ Department of Biological Sciences, Fort Hays State University, Hays, Kansas, USA

^7^ Division of Biology, Kansas State University, Manhattan, Kansas, USA

**Correspondence**

Zhe Ren, Purdue University, 1370 Lilly Hall of Life Sciences, 915 Mitch Daniels Blvd, West Lafayette, Indiana, USA, 47907

Email address: ren256@purdue.edu

**Appendix S9 Site-level summary of biomass and plant diversity (mean ± SE) along a rainfall gradient.**

| Location | Above ground live biomass (g m^-2^) | Species Richness  (number of species m^-2^) | Phylogenetic Diversity  (*p-ses*MNTD) | Functional Diversity  (*f-ses*MNTD) |
| --- | --- | --- | --- | --- |
|  | Mean ± SE | | | |
| Driest site (Colby, KS) | 235 ± 21.4 | 6.62 ± 0.37 | 0.87 ± 0.13 | -1.59 ± 0.08 |
| Dry site (Hays, KS) | 634 ± 44.1 | 6.25 ± 0.32 | 0.43 ± 0.11 | -1.09 ± 0.12 |
| Mesic site (Manhattan, KS) | 578 ± 38.3 | 7.95 ± 0.42 | 0.74 ± 0.12 | -1.2 ± 0.12 |
| Wettest site (Carbondale, IL) | 912 ± 75.1 | 12.7 ± 0.9 | 0.02 ± 0.15 | -1.29 ± 0.12 |

**Appendix S10 Site-level cross-validated root mean square error (RMSE) for biodiversity metrics along a rainfall gradient, reported to quantify the predictive accuracy of the boosted regression tree models.**

| **Location** | **Root Mean Square Error (RMSE)^*^** | | |
| --- | --- | --- | --- |
|  | **Species Richness**  **(number of species m^-2^)** | **Phylogenetic Diversity**  **(*p-ses*MNTD)** | **Functional Diversity**  **(*f-ses*MNTD)** |
| **Driest site (Colby, KS)** | 5.12 | 0.42 | 0.43 |
| **Dry site (Hays, KS)** | 4.93 | 0.75 | 0.76 |
| **Mesic site (Manhattan, KS)** | 6.64 | 0.74 | 0.75 |
| **Wettest site (Carbondale, IL)** | 12.17 | 1.01 | 0.87 |

* RMSE is reported as a measure of predictive accuracy because boosted regression trees optimize prediction error rather than variance explained, and cross-validated R² values can be unstable or misleading for such models.
